# All-in-One Process for Mass Production of Membrane-Type Carbon Aerogel Electrodes for Solid-State Rechargeable Zinc-Air Batteries

**DOI:** 10.3390/membranes12121243

**Published:** 2022-12-08

**Authors:** Hye-Rin Jo, Seung-Hee Park, Sung Hoon Ahn

**Affiliations:** Department of Bio-Chemical Engineering, Chosun University, 309 Pilmun-daero, Dong-gu, Gwangju 61452, Republic of Korea

**Keywords:** cellulose, carbon aerogel, zinc-air batteries, mass production

## Abstract

This study presents a mass-production process for conductive carbon membrane-type sponge electrodes derived from recyclable cellulose biowaste. It includes an all-in-one hydrogel fabrication process for mass production, which significantly shortens the complex and expensive process for the conventional process of catalytic electrodes based on conductive supporting substrates such as the gas diffusion layer (GDL). The presence of pre-adsorbed melamine powder in the all-in-one hydrogel induces internal diffusion of the gaseous reactant for the uniform growth of carbon nanotubes (CNTs) onto the sponge-like porous carbon aerogel with a relatively thick and tortuous pore structure, thereby providing the electrochemical properties and mechanical strength simultaneously required for the air electrodes of rechargeable and quasi solid-state zinc-air batteries.

## 1. Introduction

Demand for electrochemical conversion and storage systems is continuously increasing, including fuel cells [1], batteries [2], and the electrochemical conversion process from naturally abundant resources such as water, carbon dioxide, and ammonia to value-added chemicals [3,4,5,6]. Accordingly, the importance of highly efficient catalytic electrode design is increasing. In particular, in electrochemical reactions using gas molecules of oxygen, nitrogen, and carbon dioxide as reactants, the gas diffusion layer (GDL) requires a three-dimensional (3D) hierarchical pore structure for facile mass transfer and further exposure of accessible active sites [7,8]. Zinc-air batteries (ZABs) are one of the emerging secondary batteries due to their high theoretical energy density of ~1046 Whkg^−1^ under human-friendly aqueous alkaline operating conditions [9], and air cathodes are typically fabricated by dispersing a catalyst material onto GDL, usually made of carbon nanofiber paper.

Conductive membrane-type carbon sponge aerogels are regarded as promising platforms as air cathodes for rechargeable zinc-air batteries (ZABs). In order to ensure multi-functional catalytic activity for both Oxygen evolution reaction (OER) and oxygen reduction reaction (ORR), transition metal- and nitrogen-species should be properly introduced into the carbon aerogel on an atomic scale [10,11]. Although the most effective catalytic materials for ORR and OER are noble metal-based catalysts such as platinum (Pt) and iridium (Ir), respectively, non-noble metal-based catalysts have made significant progress in securing multi-functional activity [12,13]. Among them, carbon nanomaterials derived from metal-organic framework, particularly zeolitic imidazolate frameworks (ZIFs), exhibit very good ORR activity compared to Pt/C catalysts and moderate OER performance, and have been applied as air cathodes for rechargeable ZABs [14,15,16]. However, these powdery catalyst nanomaterials are prone to agglomeration in the annealing process for carbonization, and redispersion into ink solutions is difficult for deposition into GDL. Furthermore, the uniform deposition onto GDL with deep, tortuous pore walls with 3D structures is still challenging and requires further studies. In addition, three-dimensional (3D) pore structure plays a pivotal role, because the charge/discharge process can take place over three phases of the gas-liquid-solid interfaces. In conventional air cathodes composed of densely packed structure, the mass and electron-transfer can be limited only to the surface of electrodes [17,18,19].

Covering 3D electrodes with powdered nanomaterials is another key problem in membrane-type electrode fabrication, and the non-uniformity of catalytic nanomaterials usually results in reduced catalytic activity and overloading of the expansive catalyst materials. In order to solve this problem, there has been reported a use for self-supporting electrodes in which catalyst nanomaterials are grown directly on substrates with 3D structures such as nickel foam, and GDL (i.e., carbon cloth, carbon nanofiber paper, graphite felt) [20,21,22]. This approach not only allows for better catalytic activity, but also shortens the complex electrode manufacturing process. However, the growth of nanomaterials on 3D substrates can be limited by mass diffusion of the reactants. In general, carbon nanotubes (CNTs) can be grown on a 3D substrate through chemical vapor deposition (CVD) in which a gaseous carbon reactant was flowed during the annealing process and catalyzed by transition metal species for growth of CNTs [23,24]. Recently, it has been reported that melamine and some transition metals such as cobalt, nickel, and iron can be catalyzed to grow many nitrogen-doped CNTs [25]. In particular, metal-encapsulated CNTs generated due to the coexistence of transition metal nanoparticles, clusters, and single-atom active sites showed excellent multifunctional activity toward HER, OER [26], and ORR [27]. However, the mass production of carbon electrodes is limited in terms of the size of the substrate, because the internal-diffusion of the gas reactants is limited in the tortuous porous matrix of the 3D structure. Therefore, the introduction of a simple but mass-produced synthesis method can spur the development of catalyst electrodes based on non-noble metal catalysts.

In this study, we introduce an all-in-one hydrogel synthesis process which can mass-produce a self-supporting carbon sponge aerogel with CNTs uniformly grown on the surface. All-in-one hydrogels can be prepared by simply physically mixing transition metal species, melamine, and two different building units. As-prepared carbon aerogel was directly applied as a catalytic air cathode of ZAB with good multifunctional activity and good mechanical properties as a self-supporting electrode, completely eliminating the need for a supporting substrate and generation of interfacial resistance. This study presents a novel mass-production process of carbon aerogel electrodes with multi-functional catalytic activity for solid-state rechargeable zinc-air batteries.

## 2. Materials and Methods

### 2.1. Preparation of Membrane-Type Carbon Sponge Aerogel Electrodes

Firstly, 3 g of cobalt nitrate hexahydrate was mixed with 6 g of 2-methylimidazole in 500 mL of methanolic solution and magnetically stirred overnight. ZIF-67 powder was collected by centrifugation and washed with methanol three times. To prepare the all-in-one hydrogel solution, 1.0 g of laboratory tissue (WypAll^®^ wiper), 1.5 g of ZIF-67 powder, and 8 g of melamine were added into 50 mL of graphene oxide solution (~0.5 wt.%, Grapheneall, Siheung, Republic of Korea). The all-in-one solution was homogenized at 10,000 rpm for 10 min, and then poured into 8 pieces of silicon molds (2.5 × 2.5 cm), and then freeze-dried for 3 days. Finally, carbon aerogel sponges were obtained by an annealing process at 800 °C for 3 h with a ramping rate of 5 °C min^−1^ (hereafter denoted as CS@Co@CNTs-internal). Typically, 8 pieces of carbon aerogels can be obtained by one batch, and the carbon aerogel can be easily cut into small pieces using a razor blade.

For comparison, control samples were prepared by a similar synthetic process without an addition of melamine. The obtained melamine-free samples were annealed at 800 °C for 3 h with a ramping rate of 5 °C min^−1^ to prepare CNTs-free carbon aerogel (hereafter denoted as CS@Co). In addition, other control samples were also prepared by introducing melamine powder in an annealing process. Then 8 g of melamine powder was placed in an alumina boat, positioned at a distance of ~2 cm upstream from the alumina boat with the sample, followed by an annealing process at 800 °C for 3 h with a ramping rate of 5 °C min^−1^ (hereafter denoted as CS@Co@CNTs-external). The as-prepared carbon aerogels were directly used as catalytic electrodes without any further treatments.

### 2.2. Preparation of Conventional Air Electrodes with Commercial Noble Metal Catalysts

Benchmarking noble metal catalysts were also prepared with commercial Pt/C and Ir/C catalysts (20 wt.% on Vulcan XC-72, Naracelltech, Seoul, Republic of Korea). The conventional catalytic electrodes were prepared by deposition of catalytic ink solution with powdery catalysts (20 mg Pt/C or Ir/C dispersed into 950 μL isopropyl alcohol and 50 μL of 5 wt.% Nafion solution) onto carbon paper substrate (Avcarb P75T, Ballard, Burnaby, Canada) with a loading amount of ~4 mg cm^−2^.

### 2.3. Electrochemical Analysis

Electrochemical studies were conducted on a dual-channel electrochemical workstation (ZIVE BP2A, WONATECH, Seoul, Republic of Korea). The bi-functional electrocatalytic activity toward HER and OER was evaluated in 1 M KOH solution with three-electrode configuration with carbon aerogel or conventional electrodes as working electrodes, mercury-mercury oxide electrode filled with 1 M NaOH solution as reference electrode, and graphite rod as counter electrode, respectively. The linear sweep voltammetry (LSV) curves were recorded with a scan rate of 1 mV s^−1^. Nyquist plots were conducted by electrochemical impedance spectroscopy (EIS) with the frequency range from 1 MHz to 0.1 Hz with an amplitude perturbation of 5 mV. The ORR activity was evaluated with rotating ring-disk electrode (RRDE) experiments in O_2_-saturated 0.1 M KOH solution at a rotating rate of 1600 rpm with a scan rate of 1 mV s^−1^. The catalytic film on glassy carbon electrode was prepared by drop-casting of ~20 μL of ink solution (10 mg of catalyst dispersed into 950 μL isopropyl alcohol and 50 μL of 5 wt.% Nafion solution) on glassy carbon electrode from Pine instruments. The electron transfer number and hydrogen peroxide yield were calculated by the following equations:

(1) $$n=\frac{4I_{d}}{I_{d}+I_{r}/N}$$

(2) $${\%H}_{2}O_{2}=\frac{2I_{r}/N}{I_{d}+I_{r}/N}\times 100\%$$

where I_d_ is a ring current density, I_r_ is a ring current density, n is the electron transfer number, and N is the collection efficiency of 0.37. The recorded potential versus mercury-mercury oxide reference electrode was converted to potential versus reversible hydrogen electrode (V_RHE_) by the following Nernst equation:

(3) $$V_{\mathrm{RHE}}=V_{\mathrm{record}}+0.059\times \mathrm{pH}+V_{\mathrm{Hg}/\mathrm{HgO}}$$

The durability test of catalysts toward OER was conducted with chronopotentiometry technique in 1 M KOH electrolyte for 48 h at 10 mA cm^−2^. The accelerated durability test of catalysts toward ORR was conducted in RDE experiments in O_2_-saturated 0.1 M KOH electrolyte in the potential range from 0.2 V to 0.8 V_RHE_ for 5000 cycles.

### 2.4. Preparation and Evaluation of Rechargeable Zinc-Air Batteries

The rechargeable ZABs were fabricated based on zinc foil with ~0.5 mm thickness as zinc anode, sodium polyacrylate (PANa) hydrogel as solid-state gel electrolyte, and carbon aerogel or conventional electrodes as air cathodes. The gel electrolyte was prepared by following the literature [28]. The polymerized hydrogel membrane was saturated with 6 M KOH and 0.2 M zinc acetate dihydrate solution for 72 h until it became a transparent hydrogel and was applied to solid-state zinc-air batteries. The cyclic performance of ZABs was evaluated with the 12 channel battery test system (WBCS3000, Wonatech, Seoul, Republic of Korea), and a current density of 5 mA cm^−2^ was applied to the charge and discharge cycling test, with 300 s of time period.

### 2.5. Characteristics

The morphology of carbon sponge aerogels was studied with a field-emission scanning electron microscope (FE-SEM, Gemini 500, ZEISS, Jena, Germany) of the Center for Research Facilities at the Chonnam National University and ultra-high-resolution FE-SEM (Verios 5 UC, Thermo Fisher Scientific, Waltham, WA, USA). The N_2_ adsorption and desorption isotherm curves of carbon aerogel were obtained on BELSORP-max (MicrotracBEL Corp, Osaka, Japan), and Brunauer-Emmett-Teller (BET) surface area and pore size distribution was calculated based on the non-linear density functional theory (NLDFT) model. The detailed morphology and nanostructure of carbon aerogels was observed with transmission electron microscopy (TEM, JEM-2100F, JEOL, Tokyo, Japan) and aberration-corrected high-angle annular dark-field scanning transmission electron microscopy (HAADF-STEM, JEM ARM 200F, JEOL, Tokyo, Japan). X-ray diffraction (XRD) was carried out with X’Pert PRO diffractometer. X-ray photoelectron spectroscopy (XPS) was conducted with K-ALPHA (Thermofisher, Waltham, WA, USA). Raman spectrums were obtained by a laser Raman spectrophotometer (NRS-5100).

## 3. Results & Discussion

**Figure 1:**
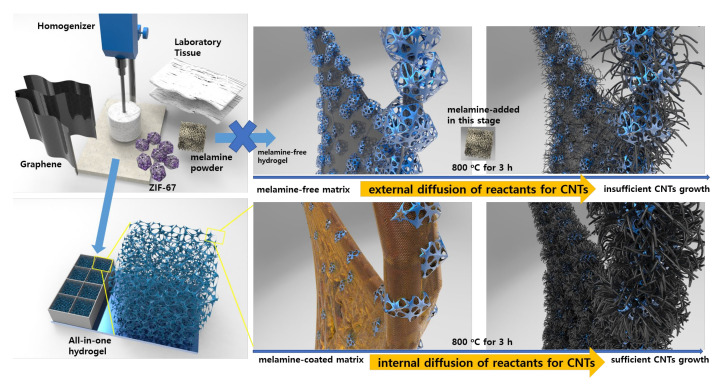
Schematic illustration of the synthetic process from an all-in-one hydrogel solution to a carbon aerogel sponge electrode.

As shown in Figure 1, all of the required components such as graphene oxide, laboratory tissue, ZIF-67 powder, and melamine were added to deionized water, and mechanically mixed with homogenizer, and this viscous solution was poured into a silicon mold to prepare 8 pieces of “all-in-one” hydrogels. In detail, the tissue was homogenized and separated into individual cellulose microfibers to make a one-dimensional (1D) skeleton, and then uniformly wrapped with two-dimensional (2D) graphene oxide layers attributed to good hydrophilic-hydrophilic interactions. Attributed to this, indicating the good mechanical strength from interconnected 1D/2D structure, the resultant hydrogel and/or carbon sponge was easily cut into smaller pieces with a razor blade. Similarly, hydrophilic ZIF-67 nanocrystals were evenly distributed and wrapped with 2D graphene layers. Finally, melamine was introduced to an “all-in-one” solution, which induces the internal diffusion of ammonia gas and provides carbon and nitrogen sources for uniform solid-state growth of CNTs on the surface of ZIF-67 nanocrystals. Typically, several pieces of hydrogels with a size of ~2.5 × 2.5 cm^2^ were obtained after the freeze-drying process, and were directly pyrolyzed at 800 °C for 3 h for carbonization and in-situ growth of CNTs in order to prepare several pieces of carbon sponges. As shown in the photograph images of Figure S1 in Supplementary Materials, the dried hydrogels slightly decreased in size after the annealing process, and changed from bright blue to completely black, indicating a high degree of graphitization mainly due to the growth of CNTs. In this study, the presence of the adsorbed melamine in “all-in-one” hydrogel is pivotal, which induces the “internal diffusion” of reactants for solid-state growth of CNTs by providing carbon and nitrogen sources in the carbon sponge.

In previous studies, transition metal species such as nickel, cobalt, and iron can react with carbon sources such as melamine during an annealing process, which catalyze the growth of CNTs encapsulating metal nanoparticles [25,29,30]. In addition, ammonia gas generated in situ from the sintering process can further etch transition metal nanoparticles into clusters and single atoms and generate a number of metal-nitrogen-carbon active sites in CNTs. In this study, the sufficient growth of CNTs onto the surface of the carbon sponge is essential to utilize Co-N-C active sites towards a multi-functional electrochemical reaction such as HER, OER, and ORR. However, the mass transfer of vaporized reactants for growth of CNTs into a three-dimensional (3D) porous structure of carbon sponge can be limited by mass transfer. Take into consideration the multiple steps of mass-diffusion on porous structure and tortuous paths in catalyst, including the external- and internal diffusion. The presence of a melamine-coated matrix directly provides the reactants to ZIF-67 nanocrystals, which is the main reaction center for growth of CNTs. As a control group, hydrogels without melamine powder were also prepared, and melamine powder was introduced in the annealing process as reported in other literature. In this case, the diffusion of the vaporized melamine-derived gas reactant is limited to the surface of the porous carbon sponge, indicating the external diffusion, and the number density of CNTs grown on the carbon membrane electrode surface is relatively low.

**Figure 2:**
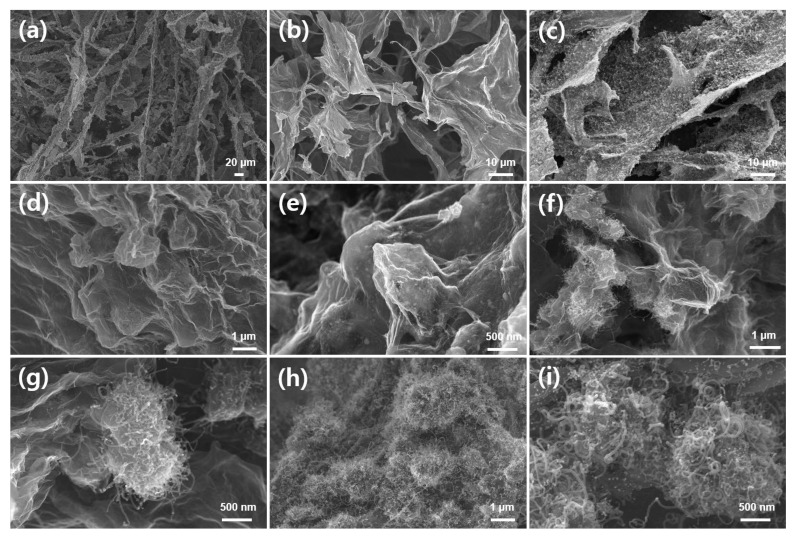
SEM images of (**a**,**b**) carbon sponge (CS), (**c**,**d**) CS@Co, (**e**,**f**) CS@Co@CNTs-external, (**g**–**i**) CS@Co@CNTs-internal.

FE-SEM images in Figure 2 show the morphology of membrane-type carbon sponge electrodes after the annealing process. As shown in Figure 2a,b, carbon sponge (CS) is comprised of 1D carbon microfiber wrapped with 2D graphene layers. By the introduction of ZIF-67 into CS (Figure 2c–e), it was observed that ZIF-67 derived nanocrystals were wrapped by graphene layers, and uniformly dispersed onto the whole surface of carbon sponge samples. The wrinkles in graphene layers were also observed in Figure 2d,e. The introduction of melamine powder during the annealing process induces the growth of CNTs anchoring on ZIF-67 derived nanocrystals (Figure 2f,g). By comparison, the presence of adsorbed melamine in all-in-one hydrogel induces the uniform growth of CNTs on the whole carbon matrix of CS@Co@CNTs-internal, as shown in Figure 2h,i. The number density as well as the length of CNTs of CS@Co@CNTs-internal were relatively higher than those of CS@Co@CNTs-external. Note that the growth of CNTs was localized around ZIF67-derived nanocrystals for CS@Co@CNTs-external, whereas uniform growth of CNTs was observed over the entire area, including the graphene matrix for CS@Co@CNTs-internal (Figure S2a,b). The uniform growth of CNTs for CS@Co@CNTs-internal indicates that the ZIF67-derived Co species diffused into the entire area of the carbon sponge during the annealing process. Due to the close contact between the pre-adsorbed melamine and ZIF67, the ammonia gas generated in situ during the annealing process further etches and disperses the Co species into nanoclusters, promoting the even growth of CNTs. For CS@Co@CNTs-internal, the amount of melamine was also systematically controlled, and as shown in Figure S3a,b, reducing the amount of melamine by half (~4 g) resulted in the local growth of CNTs only around ZIF67-derived nanocrystals, and the presence of CNTs in the graphene matrix was barely observed (Figure S4a,b). In addition, when 4 g of melamine was additionally added in the annealing process to induce both external and internal diffusion (hereafter denoted as CS@Co@CNTs-both), the growth of dense CNTs clusters was observed in the entire region of the sample (Figure S5a–d).

**Figure 3:**
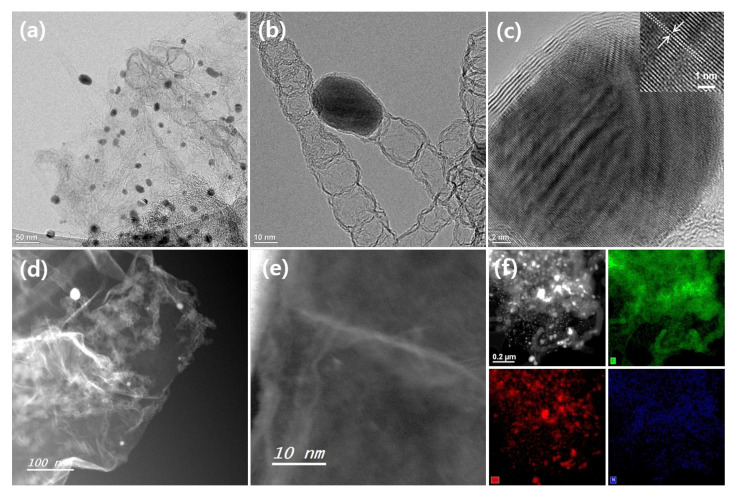
(**a**,**b**) TEM, (**c**) HR-TEM, (**d**) STEM, and (**e**) atomic-scale HAADF-STEM images, (**f**) STEM and corresponding C, Co, N elemental mapping images of CS@Co@CNTs-internal.

**Figure 4:**
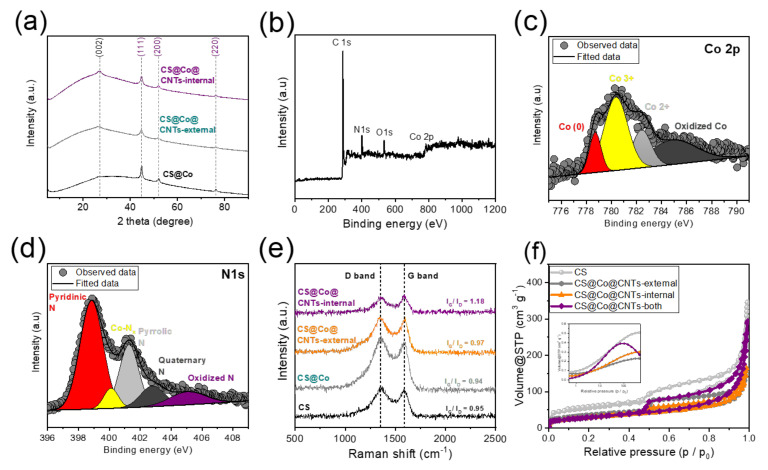
(**a**) XRD patterns of as-prepared carbon sponge electrodes, (**b**) XPS survey spectrum, fine (**c**) Co2p, (**d**) N1s spectrums of CS@Co@CNTs-internal. (**e**) Raman spectrum, (**f**) N₂ adsorption-desorption isotherm curves of carbon sponge electrodes (inset image in Figure 4f represents the pore size distribution).

The detailed nanostructure and the presence of atomic-scale Co sites in carbon matrix was observed in TEM (Figure 3a–c) and high-angle annular dark field STEM (HAADF-STEM) images (Figure 3d,e) for CS@Co@CNTs-internal. As shown in Figure 3a and Figure S6a,b, CNTs composed of bamboo-like nodules anchored on a carbon matrix, with metal nanoparticles encapsulated with graphene layers at the tips of the CNTs (Figure 3b). As shown in Figure 3c, the metal nanocrystals were encapsulated by the graphene layers, and d-spacing of 0.205 nm assigned to (001) facet of metallic Co in the lattice fringe was confirmed in the inset image of Figure 3c. As shown in Figure 3d, many CNTs were anchored on the surface of graphene layers, and atomic Co sites were observed in CNT nodules (Figure 3e). The uniform distribution of C, Co, and N elements were confirmed by energy-dispersive X-ray spectroscopy (EDX) mapping images in Figure 3f. TEM images of CS@Co@CNTs-both (internal&external) are shown in Figure S7 and exhibit a morphology similar to that of CS@Co@CNTs-internal. In the previous studies, the co-existence of Co nanoparticles, clusters, and single atoms is proven to efficiently catalyze the multiple electrochemical reactions [29,30]. Thin film-type X-ray diffraction patterns of membrane-type carbon sponge electrodes were illustrated in Figure 4a. The broad peak at 26° assigned to (002) plane of graphitic carbon of CS@Co was sharpened for CS@Co@CNTs-external and CS@Co@CNTs-internal, which attributed to the growth of CNTs, and the three peaks located at 44°, 51°, and 76° were indexed to (111), (200), and (220) facets of metallic cobalt (JCPDS 15-0806) for three samples, indicating the presence of metallic Co nanoparticles. As shown in Figure 4b, XPS survey spectrum of CS@Co@CNTs-internal clearly exhibited peaks of C, N, O, and Co, and the atomic ratios were 83.1, 6.5, 8.1, and 2.3 at%, respectively. The fine Co2p XPS spectrum in Figure 4c exhibits three main peaks located at 778.7, 780.3, and 782.4 eV, corresponding to metallic Co, Co^3+^, and Co^2+^, respectively [21], indicating the presence of Co nanoparticles and atomic sites that coordinate with nitrogen or carbon elements [31]. In N1s XPS spectrum (Figure 4d), four main peaks located at 398.8, 400.1, 401.3, and 402.8 eV were observed, corresponding to pyridinic N, Co-N_x_, pyrrolic N, and quaternary N, respectively. The survey spectrum, fine Co2p, and N1s spectrums of CS@Co (Figure S8a–c), CS@Co@CNTs-external (Figure S9a–c), and CS@Co@CNTs-both (Figure S10a–c) were also illustrated. For comparison, the nitrogen content was in the order of CS@Co@CNTs-both > CS@Co@CNTs-internal > CS@Co@CNTs-external > CS@Co. For CS@Co prepared without melamine, the atomic ratio of nitrogen was very low, less than 1 at%. The fine Co2p and N1s spectrums were deconvoluted with no significant difference from CS@Co@CNTs-internal. As shown in Figure 4e of Raman spectrum, the relative peak intensity of I_G_/I_D_ of CS was 0.95, which decreased slightly to 0. 94 with the introduction of Co species for CS@Co. For CS@Co@CNTs-external and CS@Co@CNTs-internal, the I_G_/I_D_ values increased to 0.97 and 1.18, respectively, due to the growth of CNTs with high graphitic layers. As shown in Figure 4f, N_2_ isotherm curves were illustrated in Figure 4f, and the specific surface area of CS (~197.1 m^2^g^−1^) was reduced by the introduction of Co nanoparticles for CS@Co@CNTs samples (~120 m^2^g^−1^). As shown, pore size distribution curves (inset image of Figure 4f), the intensities of CS@Co@CNTs-internal and CS@Co@CNTs-both are higher than those of CS@Co@CNTs-external, indicating an advantage in the facile mass transport of electrochemical reactants.

**Figure 5:**
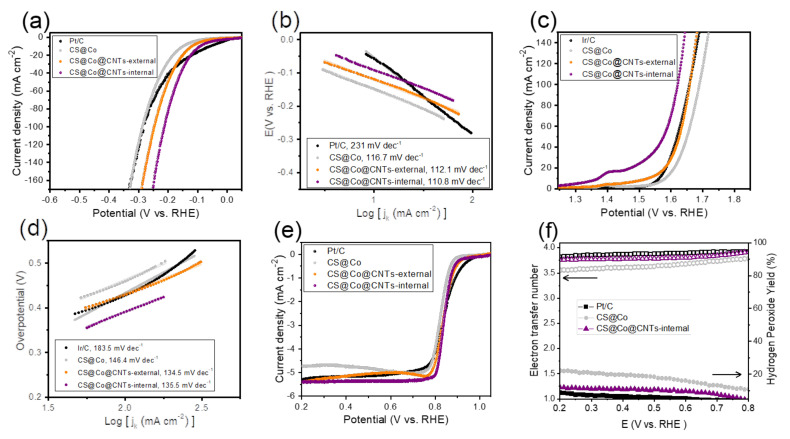
(**a**) LSV curves of as-prepared carbon sponge electrodes and Pt/C toward HER in 1 M KOH electrolyte with a scan rate of 1 mV s^−1^ and (**b**) corresponding Tafel plots. (**c**) LSV curves of as-prepared carbon sponge electrodes and Ir/C toward OER in 1 M KOH electrolyte with a scan rate of 1 mV s^−1^ and (**d**) corresponding Tafel plots. (**e**) LSV curves of samples based on RRDE experiments in O_2_-saturated 0.1 M KOH electrolyte with a scan rate of 1 mV s^−1^ and at a rotating rate of 1600 rpm. (**f**) Hydrogen peroxide yields and electron transfer numbers calculated from RRDE results.

The multi-functional catalytic activity of carbon aerogel samples and commercial Pt/C or Ir/C catalysts were evaluated with an electrochemical workstation. As shown in Figure 5a of LSV curves, CS@Co@CNTs-internal exhibits excellent HER activity in terms of the recorded overpotential value (η_j10_) of 93 mV at a current density of 10 mA cm^−2^, which is relatively smaller than that of CS@Co@CNTS-external (η_j10_ = 120.2 mV) and CS@Co (η_j10_ = 149.9 mV), and slightly higher than Pt/C (η_j10_ = 58 mV). The advantage of membrane-type carbon sponge electrodes showed particularly in high current density, and the overpotential values of CS@Co@CNTs-internal at the current density of 100 mA cm^−2^ (η_j100_) was 213 mV, which is rather smaller than that of Pt/C (η_j100_ = 287 mV). The Tafel slope values of the three carbon sponge-based samples in Figure 5b were in the range of 110.8 to 116.7 mV dec^−1^, which is much smaller than that of Pt/C (231 mV dec^−1^), indicating facile electron and mass transfer due to the porous 3D structure. The Nyquist plots were shown in Figure S11a, and CS@Co@CNTs-internal exhibited the smallest R_ct_ value among the samples, indicating the facile electron transfer in the interconnected carbon sponge matrix. It has been reported that self-supporting carbon sponge electrodes can minimize the interfacial resistance compared to conventional catalyst electrodes consisting of a powder catalyst layer on a supporting substrate such as nickel foam or carbon nanofiber paper. As shown in Figure 5c, CS@Co@CNTs-internal showed the highest OER activity with the smallest overpotential value at current density of 50 mA cm^−2^ (η_j50_ = 346.1 mV), surpassing the other samples, including commercial Ir/C catalyst. Note that the high charging current due to the redox reaction of Co species at ~1.40 V vs. RHE (V_RHE_) was observed, and we are reporting the overpotential value at relatively high current density of 50 mA cm^−2^, instead of 10 mA cm^−2^. The corresponding Tafel plots were obtained as shown in Figure 5d, and the carbon sponge-based samples again exhibited much smaller Tafel slope values (134.5~146.4 mV dec^−1^) compared to that of Ir/C of 183.5 mV dec^−1^, indicating more favorable OER kinetics. The smaller Tafel slopes of carbon sponge electrodes can be attributed to the self-supporting 3D carbon matrix, which facilitates mass diffusion of the reactants into accessible active sites. The Nyquist plots (Figure S11b) again demonstrate that the CS@Co@CNTs-internal sample has the smallest R_ct_ toward OER. The bi-functional activity of CS@Co@CNTs-both (internal and external) was also confirmed, as shown in Figure S12a,b, and there was no significant difference from that of CS@Co@CNTs-internal. The OER activities of CS@Co@CNTs-internal and CS@Co@CNTs-both were analyzed by cyclic voltammetry (CV) with a relatively slow scan rate of 0.5 mV s^−1^. As shown in Figure S12c, the forward and reverse scan curves appeared similar with no significant difference. Lastly, ORR activity of samples was evaluated in RRDE experiments, and LSV curves were obtained (Figure 5e). The half wave potential (V_half_) of CS@Co@CNTs-internal was 0.842 V_RHE_, with the limiting current density (j_lim_) of 5.38 mA cm^−2^, which is comparable to that of Pt/C (V_half_ = 0.841 V, j_lim_ = 5.36 mA cm^−2^). With similar results, the Tafel slope of CS@Co@CNTs-internal exhibited the smallest value among the samples (Figure S13). As shown in Figure 5f, the calculated CS@Co@CNTs-internal from the ring and disk currents exhibited an electron transfer number of ~4 and a hydrogen peroxide yield of less than ~10%, similar to that of the Pt/C catalyst. As shown in Figure 5f, the electron transfer number close to four and hydrogen peroxide yields of less than 10%, calculated from ring and disk currents, were similar to those of Pt/C. Note that the tri-functional activity reported in this study is one of the best among recently reported electrocatalysts [32,33,34,35,36]. Additionally, the durability of the catalysts toward both ORR and OER were investigated. As shown in Figure S14a, the recorded half-wave potential of CS@Co@CNTs-internal showed no significant change after the accelerated durability test for 5000 cycles. For OER, no obvious changes were observed in the applied potential during the chronopotentiometry test for 70 h (Figure S14b), indicating the excellent durability of CS@Co@CNTs-internal toward both OER and ORR.

**Figure 6:**
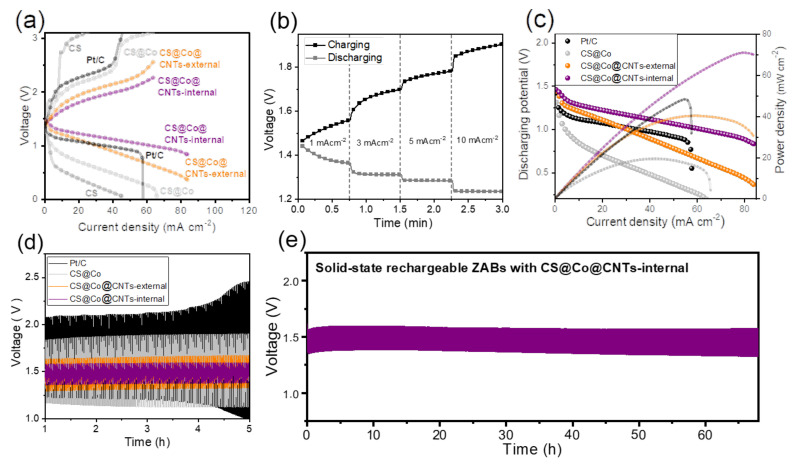
(**a**) Charge-discharge polarization curves of ZABs, (**b**) galvanostatic charge-discharge curves of ZAB with CS@Co@CNTs-internal air cathode at the different current densities. (**c**) The discharge polarization and power density curves. (**d**) Cyclic performance of ZABs. (**e**) Long-term cyclic performance of ZABs with CS@Co@CNTs-internal air cathode.

The rechargeable, solid-state zinc-air batteries (ZABs) were assembled with zinc foil as anode, carbon sponges or commercial Pt/C electrodes as the air cathodes, and PANa hydrogel as the gel electrolyte, respectively. As shown in Figure S15 of the photograph image, and in Figure 6a of polarization curves, a high open circuit voltage of ~1.44–1.48 V was observed for ZAB with CS@Co@CNTs-internal air cathode, which is higher than that of Pt/C air cathode (~1.34 V). The charge and discharge voltages of ZABs gradually differ from OCV as the current density increases, and the voltage difference was the smallest for CS@Co@CNTs-internal air cathode, reflecting the excellent bi-functional activity in practical devices. The galvanostatic charge-discharge curves of CS@Co@CNTs-internal were also shown in Figure 6b with the current densities at 1, 3, 5, and 10 mA cm^−2^, with similar potential values obtained from polarization curves. The power density of batteries was also illustrated in Figure 6c, and the peak power density of CS@Co@CNTs-internal was the highest of 70.91 mW cm^−2^, which is higher than that of Pt/C cathode (~48.31 mW cm^−2^). The cyclic performance of ZABs with an applied current density of 5 mA cm^−2^ was recorded in Figure 6d, and the voltage gap of CS@Co@CNTs-internal was only 0.22 V (1.59 V − 1.37 V), which is much smaller than that of Pt/C (2.08 V − 1.23 V = 0.85 V). Owing to the ultrahigh water uptake of PANa gel electrolyte, this solid-state rechargeable ZAB with CS@Co@CNTs-internal was well-operated at 5 mA cm^−2^ over 70 h, without significant voltage gap difference compared to the initial value (Figure 6e). Although direct comparison is difficult due to different cycling conditions, the performance values of solid-state ZAB were compared with values in other literature [37,38,39,40,41,42,43] and are summarized in Table S1. Attributed to the self-supporting 3D carbon matrix that facilitates the penetration of gel electrolyte, the voltage gap between the charge-discharge process of CS@Co@CNTs-internal was much smaller than in other studies. The durability of air cathodes after the cycling test of ZABs was evaluated by conducting SEM (Figure S16), XRD (Figure S17), and XPS (Figure S18) analysis. In SEM images, the overall nanostructure of the carbon sponge aerogel was well maintained, and anchoring CNTs were also observed for CS@Co@CNTs-internal (Figure S16d). In addition, the results of the XRD pattern and the XPS spectrums did not show a significant difference from the original, indicating that CNTs with a high graphitization degree were not severely degraded during the operation of ZAB, especially under oxidizing conditions during the charging process.

## 4. Conclusions

In this study, a mass production process of carbon sponge aerogel for the application of ZABs as air cathode was developed. Due to the porous structure and tortuous pore wall of the sponge-like aerogel electrode, the external-diffusion of the gas-phase reactants for CNT growth was limited. On the other hand, by introducing the pre-adsorption of the melamine into the all-in-one hydrogel, CNTs were sufficiently grown over the entire area of the carbon sponge through the internal-diffusion of the gas reactant, thereby satisfying both the excellent mechanical strength and multifunctional activity required for the air cathode of ZABs. As the self-supporting electrode to minimize interfacial resistance, carbon aerogel electrodes exhibited superior multi-functional activity over conventional electrodes fabricated with commercial noble metal catalysts such as Pt/C and/or Ir/C. As air cathodes for solid-state rechargeable ZABs, very small voltage gaps between the charge and discharge potentials and excellent cyclic performance have also been demonstrated.

## Data Availability

Not applicable.

## Supplementary Materials

The following supporting information can be downloaded at: https://www.mdpi.com/article/10.3390/membranes12121243/s1, Figure S1: Photograph images of freeze-dried carbon sponge samples (top) and CS@Co@CNTs samples (bottom) after an annealing process; Figure S2. Low-resolution SEM images of (a,b) carbon matrix of CS@Co@CNTs-internal; Figure S3. SEM images of (a,b) CS@Co@CNTs-internal with half amount of melamine powder (~4 g) compared to the typical process; Figure S4. SEM images of (a,b) carbon matrix of CS@Co@CNTs-internal with half amount of melamine powder (~4 g) compared to the typical process; Figure S5. SEM images of (a,b) CS@Co@CNTs-both (internal-external) with additional 4 g of melamine powder compared to the typical process; Figure S6. HAADF-STEM images of CS@Co@CNTs-internal; Figure S7. (a–d) TEM images of CS@Co@CNTs-both (internal-external); Figure S8. (a) XPS survey spectrum, fine (b) Co2p, (c) N1s spectrums of CS@Co; Figure S9. (a) XPS survey spectrum, fine (b) Co2p, (c) N1s spectrums of CS@Co@external; Figure S10. (a) XPS survey spectrum, fine (b) Co2p, (c) N1s spectrums of CS@Co@both (internal-external); Figure S11. Nyquist plots of samples toward (a) HER, and (b) OER; Figure S12. (a) LSV curves of CS@Co@CNTs-both toward (a) OER, and (b) HER, respectively. (c) Cyclic voltammetry (CV) of CS@Co@CNTs-internal and CS@Co@CNTs-both toward OER with a scan rate of 0.5 mV s^−1^; Figure S13. Tafel plots of samples toward ORR, correspond to the panel of Figure 5e; Figure S14. (a) LSV curves of CS@Co@CNTs-internal before, and after an accelerated durability test for 5000 cycles, (b) chronopotentiometry test with an applied current density of 50 mA cm^−2^; Figure S15. Photograph image of the assembled ZABs with electrochemical workstation, indicating open circuit voltage (OCV) of 1.48 V as shown in the display panel; Figure S16. SEM image of carbon-based air cathodes of (a,b) CS@Co, (c) CS@Co@CNTs-external, (d) CS@Co@CNTs-internal after cycling test of ZABs; Figure S17. XRD patterns of carbon-based air cathodes after cycling test; Figure S18. (a) XPS survey, and (b) fine Co2p, (c) N1s spectrums of CS@Co@CNTs-internal after cycling test. Table S1. Comparison of performance of solid-state ZABs with other literatures. References [37,38,39,40,41,42,43] are cited in the Supplementary Materials.
