# Supplementary material for: All-in-One Process for Mass Production of Membrane-Type Carbon Aerogel Electrodes for Solid-State Rechargeable Zinc-Air Batteries
Source: Membranes (Basel). 2022 Dec 8;12(12):1243. doi: 10.3390/membranes12121243 (PMC9785701; doi:10.3390/membranes12121243)
Supplement: Supplementary file 1 [file membranes-12-01243-s001.zip › membranes-2047982-supplementary.pdf]

All-in-one process for mass production of membrane-type carbon aerogel electrodes for solid-state rechargeable zinc-air batteries

Hye-Rin Jo, Seung-Hee Park, and Sung Hoon Ahn*

Department of Bio-chemical Engineering, Chosun University, 309 Pilmun-daero, Dong-gu, Gwangju 61452, South Korea
*Correspondence: sunghoon@chosun.ac.kr;

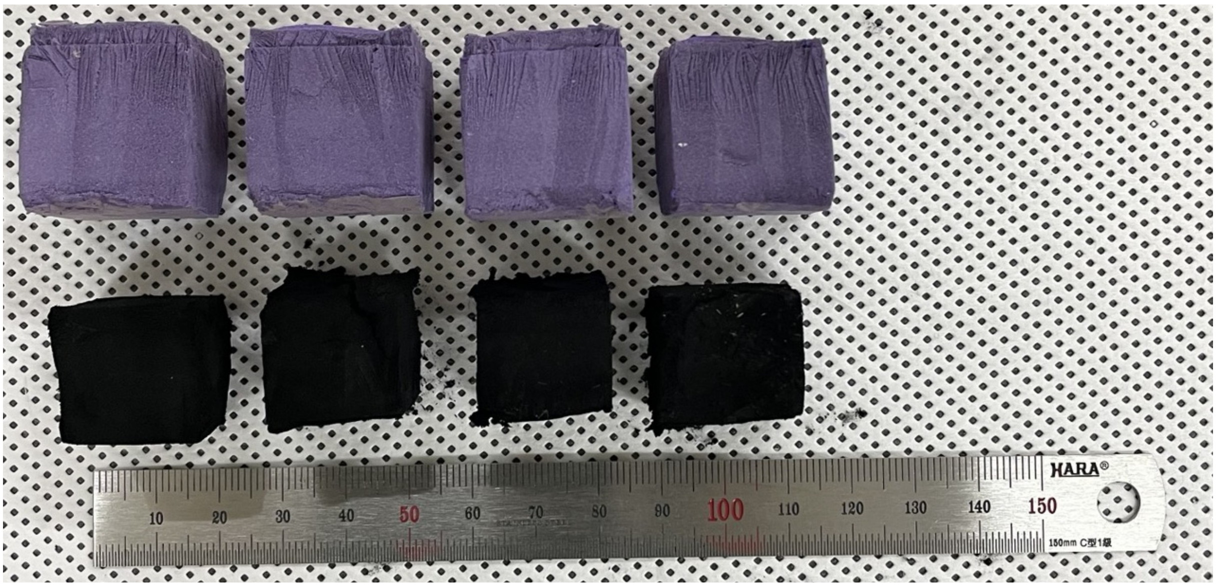

Figure S1. Photograph images of freeze-dried carbon sponge samples (top) and CS@Co@CNTs samples (bottom) after an annealing process

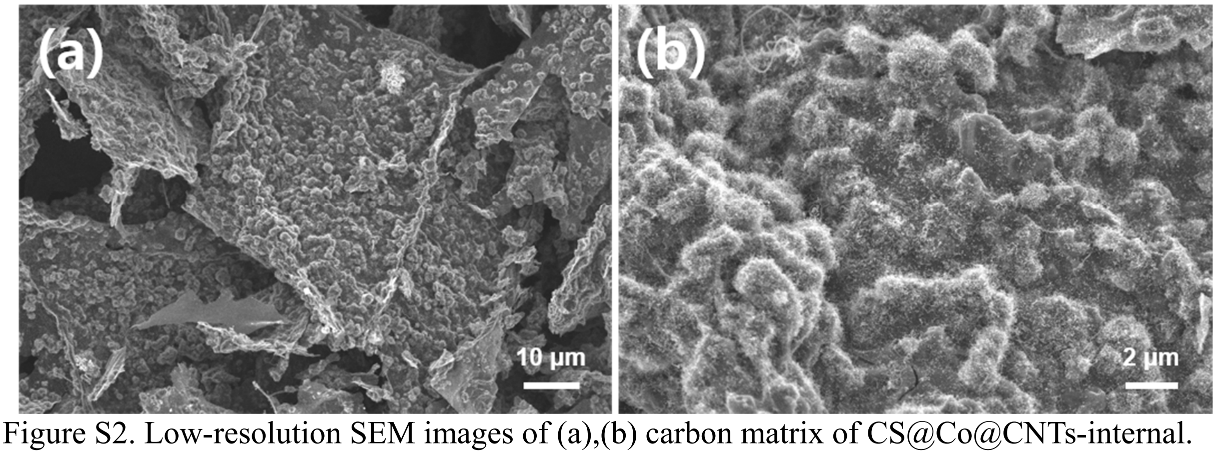

Figure S2. Low-resolution SEM images of (a),(b) carbon matrix of CS@Co@CNTs-internal.

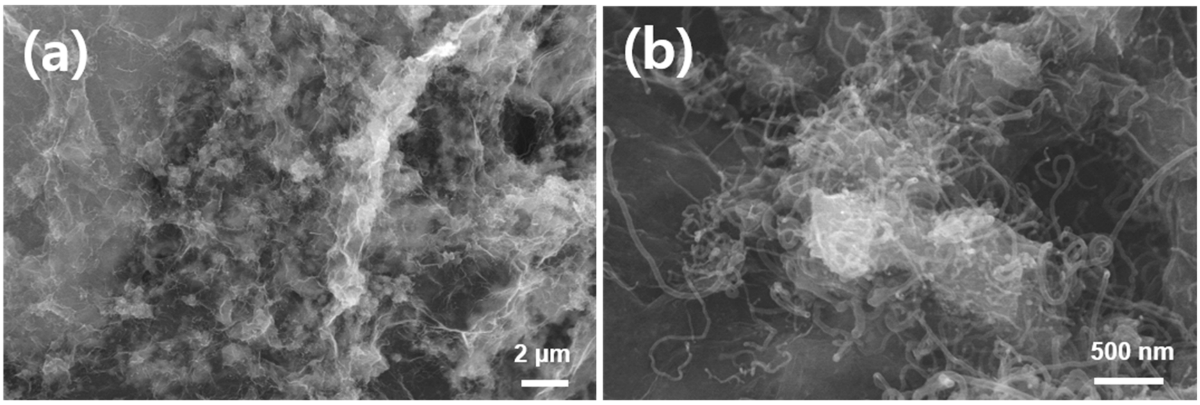

Figure S3. SEM images of (a),(b) CS@Co@CNTs-internal with half amount of melamine powder (~4 g) compared to the typical process.

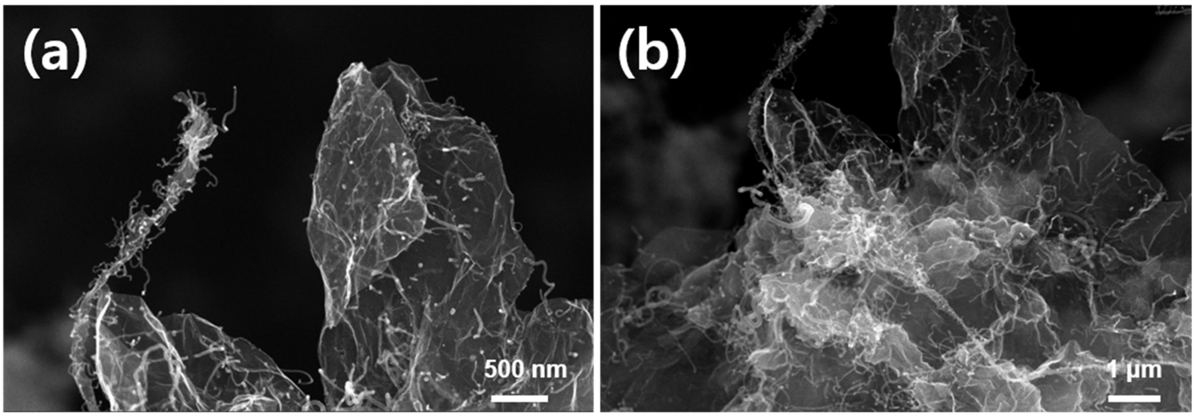

Figure S4. SEM images of (a),(b) carbon matrix of CS@Co@CNTs-internal with half amount of melamine powder (~4 g) compared to the typical process.

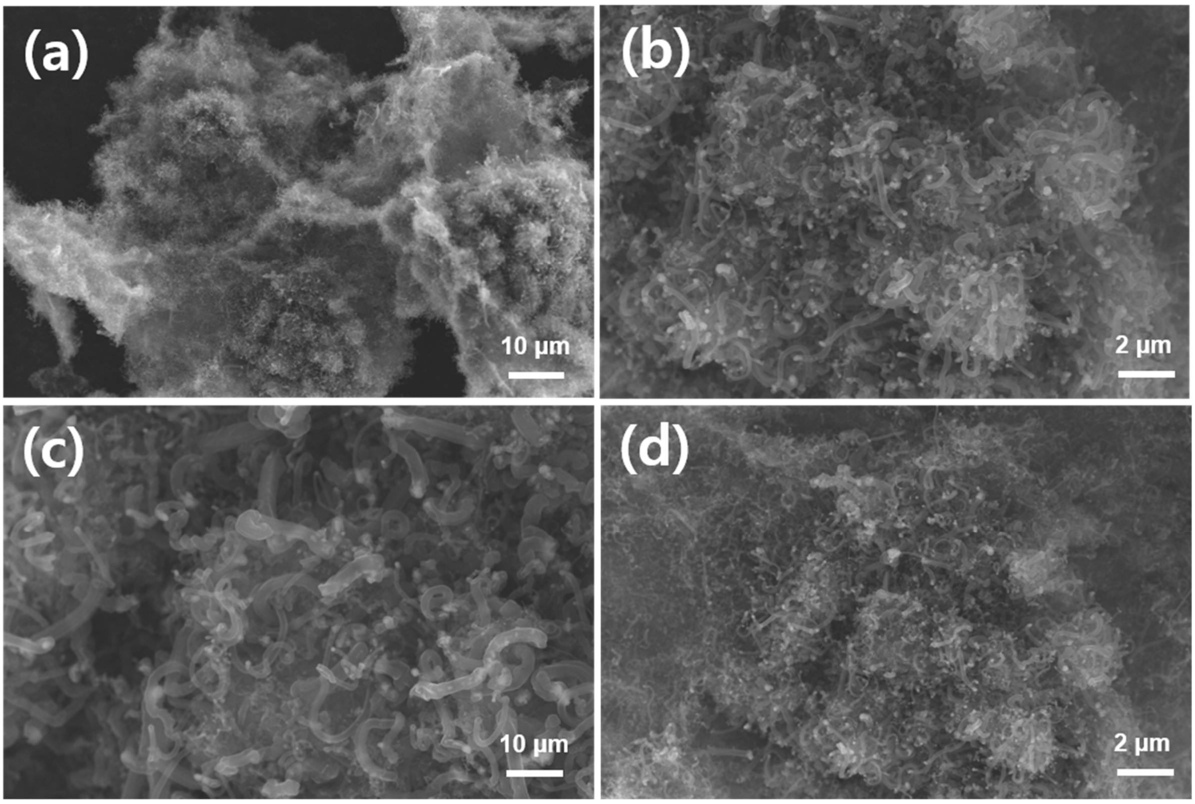

Figure S5. SEM images of (a),(b) CS@Co@CNTs-both (internal-external) with additional 4 g of melamine powder compared to the typical process.

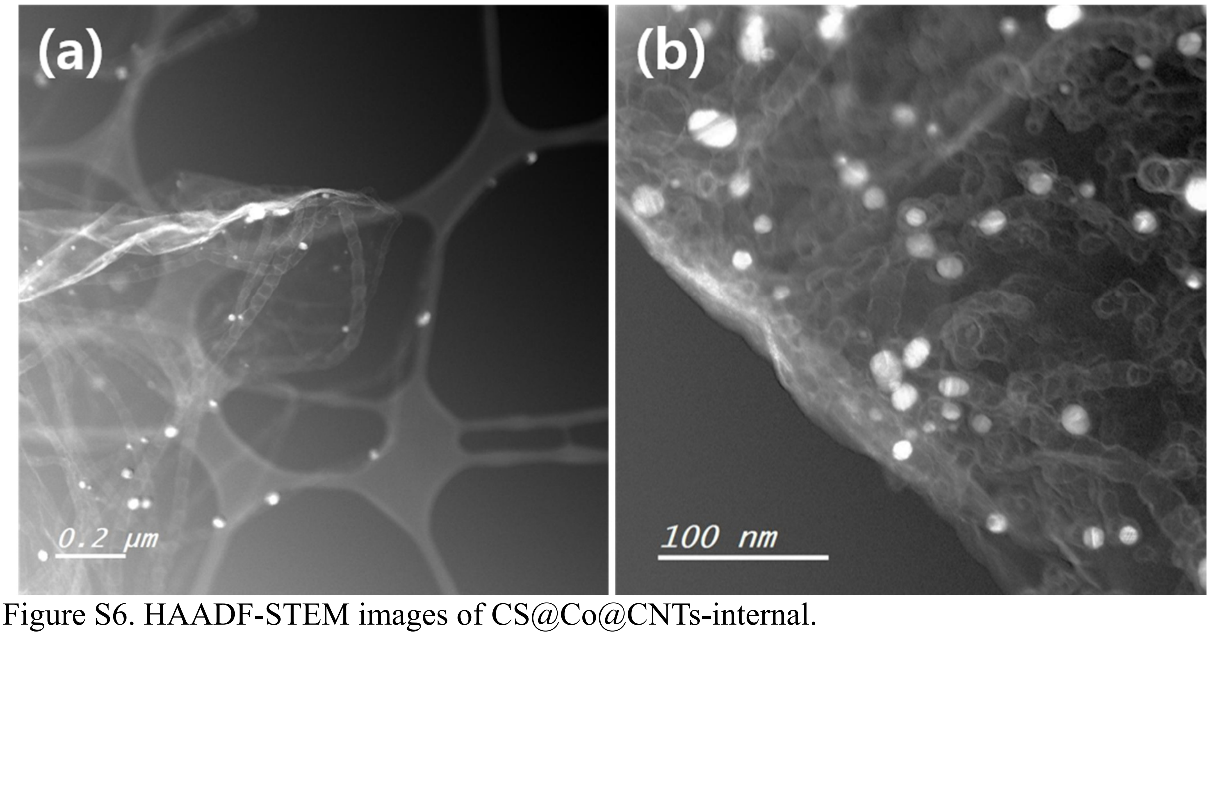

Figure S6. HAADF-STEM images of CS@Co@CNTs-internal.

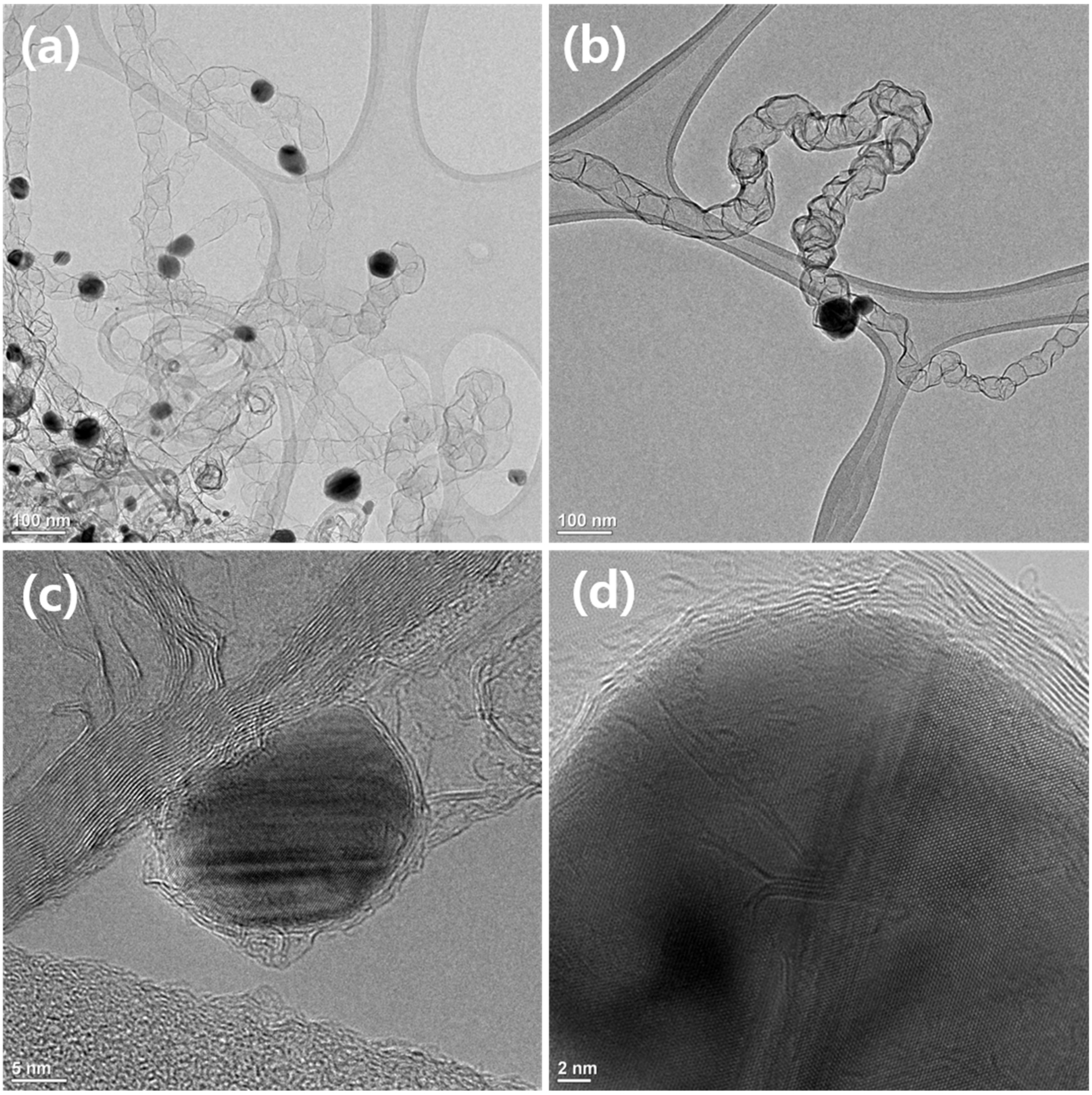

Figure S7. (a)-(d) TEM images of CS@Co@CNTs-both (internal-external)

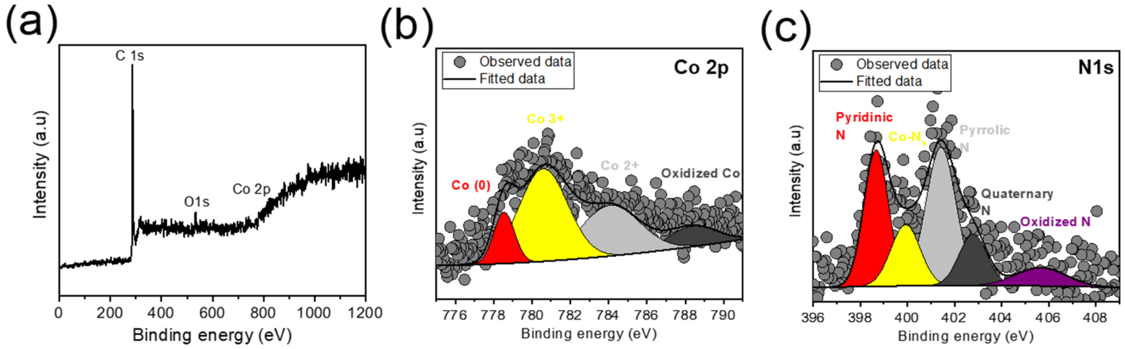

Figure S8. (a) XPS survey spectrum, fine (b) Co2p, (c) N1s spectrums of CS@Co.

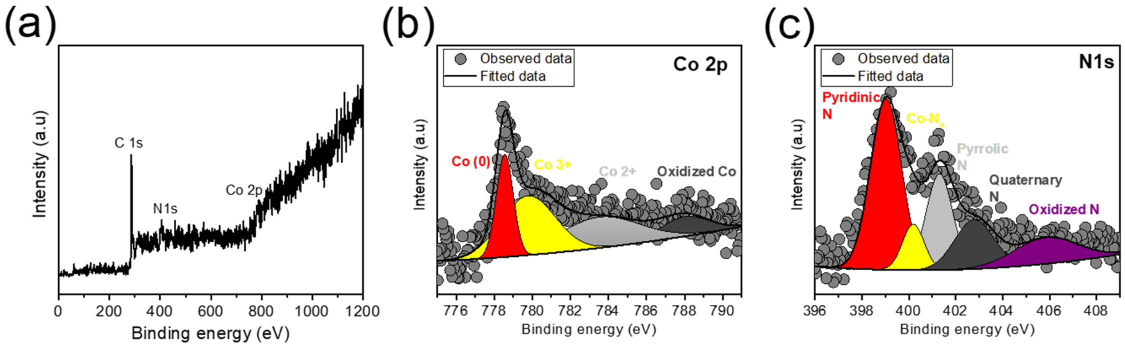

Figure S9. (a) XPS survey spectrum, fine (b) Co2p, (c) N1s spectrums of CS@Co@external.

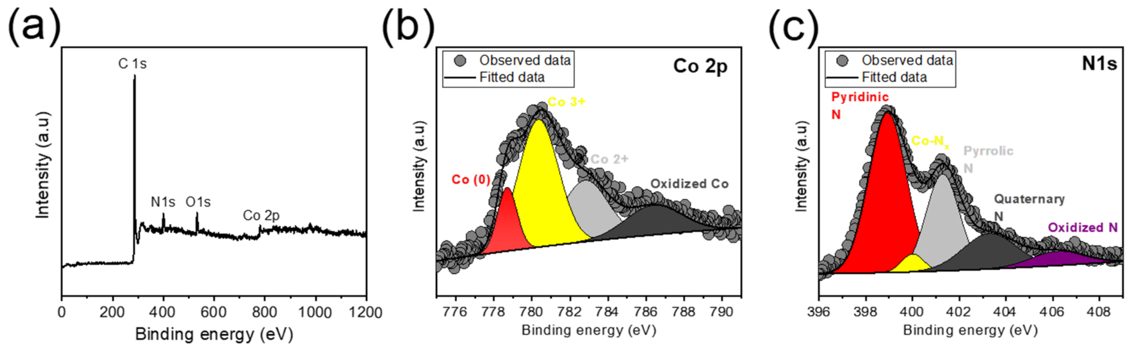

Figure S10. (a) XPS survey spectrum, fine (b) Co2p, (c) N1s spectrums of CS@Co@both (internal-external).

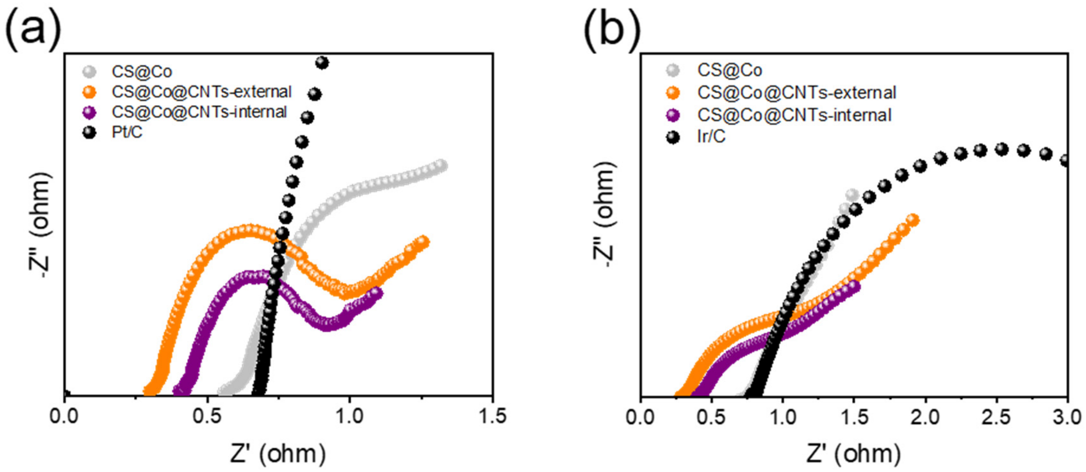

Figure S11. Nyquist plots of samples toward (a) HER, and (b) OER.

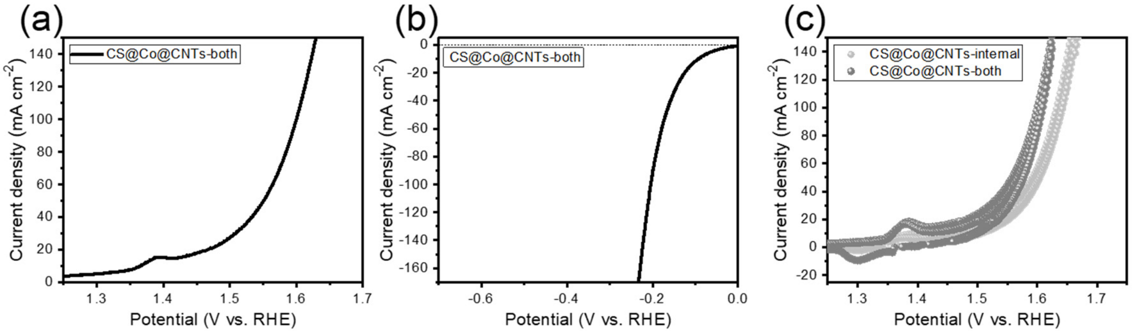

Figure S12. (a) LSV curves of CS@Co@CNTs-both toward (a) OER, and (b) HER, respectively. (c) Cyclic voltammetry (CV) of CS@Co@CNTs-internal and CS@Co@CNTs-both toward OER with a scan rate of 0.5 mV s^{-1}

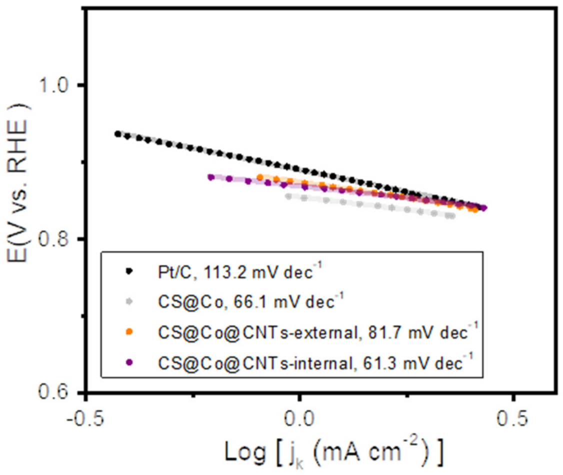

Figure S13. Tafel plots of samples toward ORR, correspond to the panel of Figure 5e.

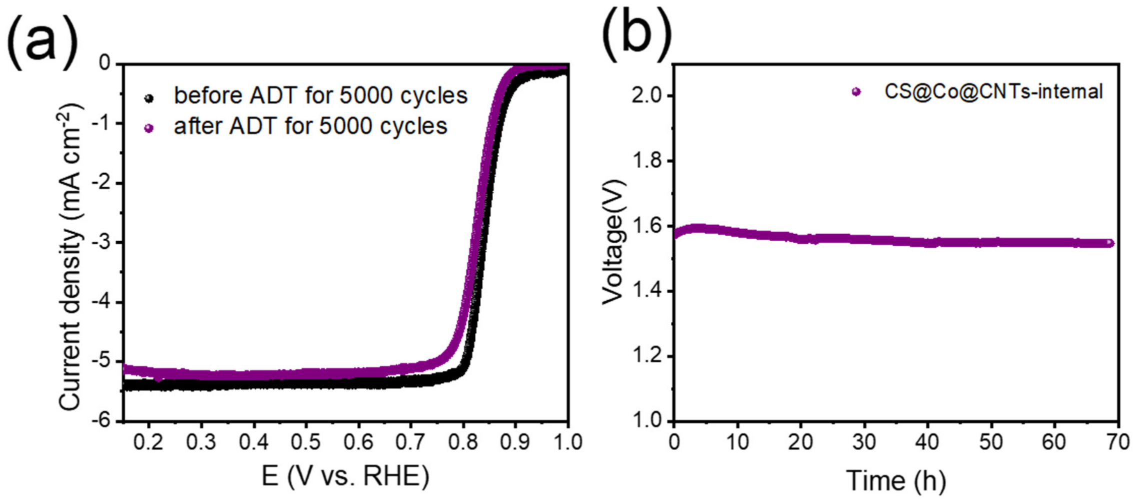

Figure S14. (a) LSV curves of CS@Co@CNTs-internal before, and after an accelerated durability test for 5000 cycles, (b) chronopotentiometry test with an applied current density of 50 mA cm^{-2}.

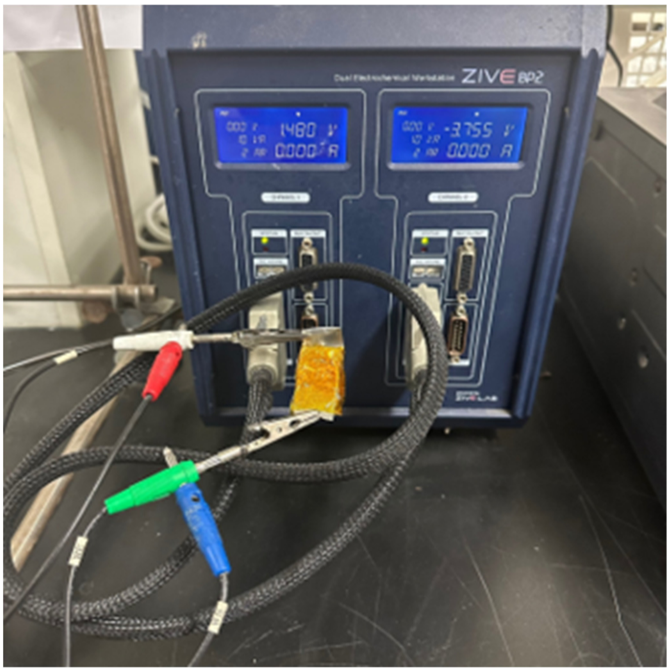

Figure S15. Photograph image of the assembled ZABs with electrochemical workstation, indicating open circuit voltage (OCV) of 1.48 V as shown in the display panel.

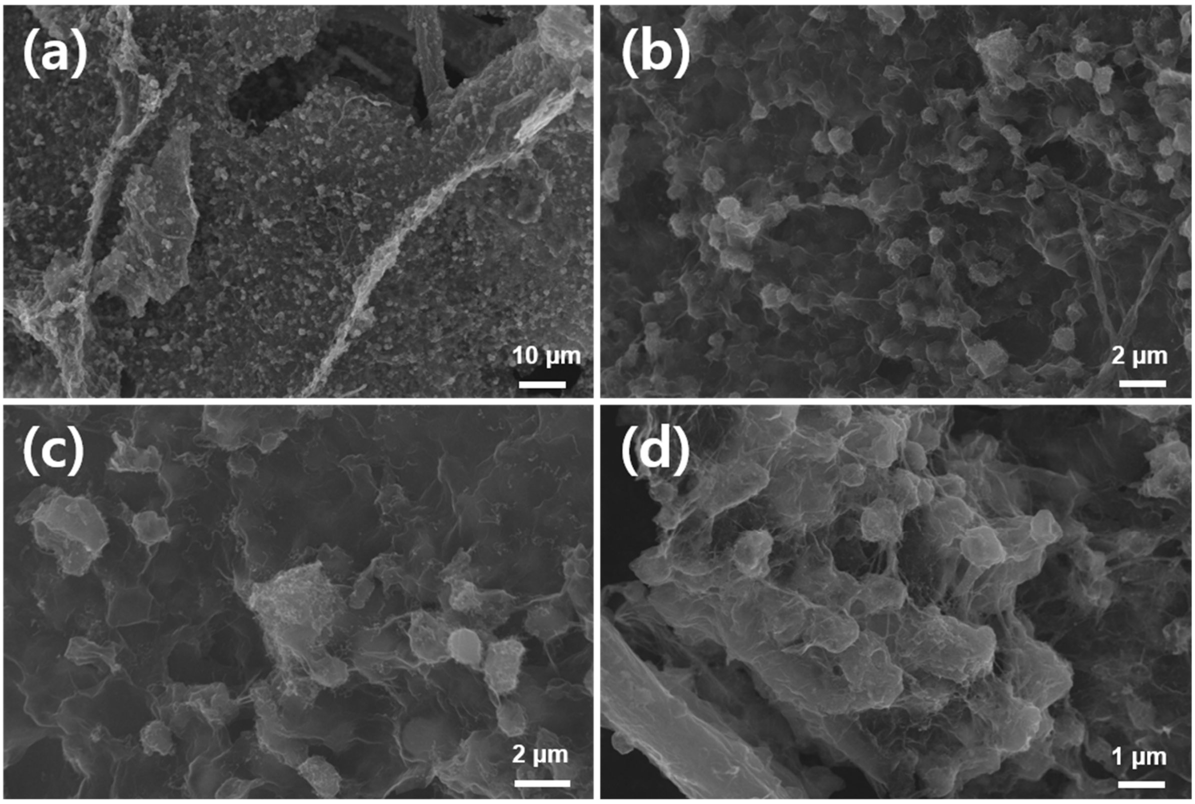

Figure S16. SEM image of carbon-based air cathodes of (a),(b) CS@Co, (c) CS@Co@CNTs-external, (d) CS@Co@CNTs-internal after cycling test of ZABs.

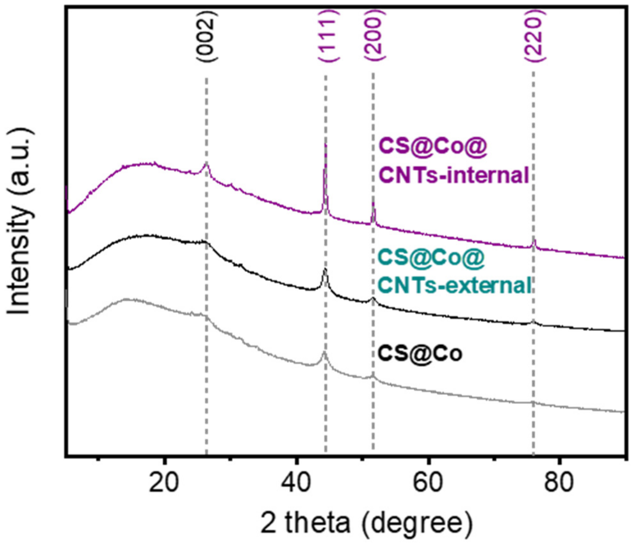

Figure S17. XRD patterns of carbon-based air cathodes after cycling test.

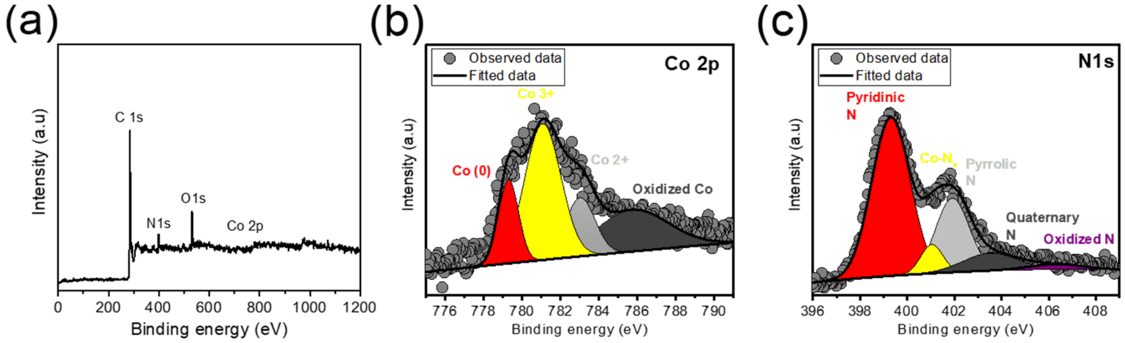

Figure S18. (a) XPS survey, and (b) fine Co2p, (c) N1s spectrums of CS@Co@CNTs-internal after cycling test.

Table S1. Comparison of performance of solid-state ZABs with other literatures.

Catalysts	Polymer gel eletrolyte	OCV (V)	Applied current density ($mAcm^{-2}$)	Voltage gap (V)	Peak power density ($mWcm^{-2}$)	Cycling time (h)	Reference number
CS@Co@CNTs-internal	PANa	1.44	5	0.22		70	this study
CNT paper	PANa	1.48	5	0.8	115	110	[40]
$Ru\text{-}RuO_2$	PVA	1.43	10	1	29	70	[37]
Co_3O_4-loaded carbon cloth	PVA with 5wt.% SiO_2	-	3	0.7	62.6	48	[41]
NiCoOH	PANa	-	2	0.68	88	160	[43]
CoN_4/NG	PVA	-	1	0.692	28	6	[42]
CS-NFO@PNC-700	PVA	1.32	1	0.662	51	40	[38]
Fe-N_x-C	PVA	1.20	10	1.27	15.8	20	[39]

[37] Wang, N.; Ning, S.; Yu, X.; Chen, D.; Li, Z.; Xu, J.; Meng, H.; Zhao, D.; Li, L.; Liu, Q.; et al. Graphene composites with Ru-RuO2 heterostructures: Highly efficient Mott–Schottky-type electrocatalysts for pH-universal water splitting and flexible zinc–air batteries. *Appl. Catal. B- Environ.* **2022**, *302*, 120838, doi:https://doi.org/10.1016/j.apcatb.2021.120838.

[38] Ramakrishnan, S.; Velusamy, D.B.; Sengodan, S.; Nagaraju, G.; Kim, D.H.; Kim, A.R.; Yoo, D.J. Rational design of multifunctional electrocatalyst: An approach towards efficient overall water splitting and rechargeable flexible solid-state zinc–air battery. *Appl. Catal. B- Environ.* **2022**, *300*, 120752, doi:https://doi.org/10.1016/j.apcatb.2021.120752.

[39] Zhao, M.; Liu, H.; Zhang, H.; Chen, W.; Sun, H.; Wang, Z.; Zhang, B.; Song, L.; Yang, Y.; Ma, C.; et al. A pH-universal ORR catalyst with single-atom iron sites derived from a double-layer MOF for superior flexible quasi-solid-state rechargeable Zn–air batteries. *Energy Environ. Sci.* **2021**, doi:10.1039/D1EE01602D.

[40] Ma, L.; Chen, S.; Wang, D.; Yang, Q.; Mo, F.; Liang, G.; Li, N.; Zhang, H.; Zapien, J.A.; Zhi, C. Super-Stretchable Zinc–Air Batteries Based on an Alkaline-Tolerant Dual-Network Hydrogel Electrolyte. *Adv. Energy Mater.* **2019**, *9*, 1803046, doi:https://doi.org/10.1002/aenm.201803046.

[41] Fan, X.; Liu, J.; Song, Z.; Han, X.; Deng, Y.; Zhong, C.; Hu, W. Porous nanocomposite gel polymer electrolyte with high ionic conductivity and superior electrolyte retention capability for long-cycle-life flexible zinc–air batteries. *Nano Energy* **2019**, *56*, 454-462, doi:https://doi.org/10.1016/j.nanoen.2018.11.057.

[42] Yang, L.; Shi, L.; Wang, D.; Lv, Y.; Cao, D. Single-atom cobalt electrocatalysts for foldable solid-state Zn-air battery. *Nano Energy* **2018**, *50*, 691-698, doi:https://doi.org/10.1016/j.nanoen.2018.06.023.

[43] Huang, Y.; Li, Z.; Pei, Z.; Liu, Z.; Li, H.; Zhu, M.; Fan, J.; Dai, Q.; Zhang, M.; Dai, L.; et al. Solid-State Rechargeable Zn//NiCo and Zn–Air Batteries with Ultralong Lifetime and High Capacity: The Role of a Sodium Polyacrylate Hydrogel Electrolyte. *Adv. Energy Mater.* **2018**, *8*, 1802288, doi:https://doi.org/10.1002/aenm.201802288.
